# Special Issue “The State of the Art in Endodontics” Part II

**DOI:** 10.3390/jcm11154511

**Published:** 2022-08-02

**Authors:** Alfredo Iandolo, Giuseppe Pantaleo, Dina Abdellatif, Massimo Amato

**Affiliations:** 1 Department of Endodontics, Faculty of Dentistry, University of Salerno, 84121 Salerno, Italy; giuseppepantaleo88@gmail.com (G.P.); maxamato1@gmail.com (M.A.); 2 Department of Endodontics, Faculty of Dentistry, University of Alexandria, Alexandria 5424041, Egypt; dinaabdellatif81@gmail.com

The long-lasting success of root canal treatment can be achieved by applying the many advancements and technologies developed during the treatment. Hence, the endodontic system is complex; the ability to perform adequate 3D cleaning after root canal shaping of this complicated system can affect the treatment outcome. Equally important is creating a total 3D obturation of the complicated endodontic system. Many clinical and histological studies demonstrate that the root canal space is formed of zones effortlessly attainable with hand and rotating files (the main root canals). Moreover, some areas are challenging to access or inaccessible (isthmuses, loops, lateral canals, ramifications, deltas and dentinal tubules; Figure 1) [1,2].

As explained above, relying only on mechanical instrumentation to clean the root canal system is ineffective to reach all the parts of the complicated endodontic system. Nevertheless, the technique leaves parts of the root canals untouched and untreated. Consequently, performing endodontic chemical cleaning (to reach accessible and inaccessible areas) is crucial. Once all areas of the endodontic system have been cleaned, they can be filled with guttapercha and sealer in the obturation step [3,4].

Researchers have worked on improving endodontic treatment in the past years.

Lately, the focus of endodontic enhancement has been on the concept of minimally invasive treatment. In fact, the conservative approach covers all the steps of root canal treatment, including the conservative access cavity, minimally invasive shaping, 3D cleaning, and 3D obturation [4].

Ordinarily, mechanical instrumentation is an essential step on which the quality of the whole root canal treatment relies. This phase completely extirpates the vital and necrotic pulp tissue, bacteria, and toxins from the main root canal.

On the other hand, the clinician must follow some rules to prepare the main canal adequately. For example, the shaping should maintain the original anatomy of the canal without any modification; particularly, the shaping must not alter the foramen. Similarly, it should prevent undue dentin reduction to avoid the risk of micro-cracks or stripping perforation.

In brief, root canal mechanical preparation is a critical step on which the quality of the whole root canal treatment counts. Furthermore, this step aims to extirpate the vital and necrotic pulp tissues, removing as many bacteria and toxins as possible from the major canal. At the same time, the root canal walls are also shaped to form a virtual space to accommodate the following step of 3D cleaning, which aims to eliminate bacteria deep in the dentinal tubules and achieve long-term success without creating possible iatrogenic errors.

Indeed, introducing empowered and modified irrigant activation techniques has rendered the achievability of conservative shaping possible and obtained outstanding results in cleaning the walls and in terms of success rates. Iandolo et al. showed that using the irrigant activation technique of intracanal heating of sodium hypochlorite followed by ultrasonic activation after conservative shaping can lead to great treatment outcomes 20.05 [5,6,7,8].

Furthermore, studies showed that minimally invasive preparations lead to various advantages. For example, less trained clinicians can perform quicker and better reproducible shaping. Additionally, it decreased the hazard of modifying the anatomy of the root canals or the apical foramen and, simultaneously, reduced the risk of developing stripping in delicate anatomies [9,10].

Conservative approaches render more consequential dentin preservation. Eventually, utilising small-sized rotary instruments of the new generation, the danger of instrument separation becomes nearly zero in the martensitic phase [9,10,11,12].

In general, many factors can alter the rate at which a chemical reaction happens. For example, it can increase with the increase in temperature, pressure, excitation and concentration. When it comes to the conditions of the root canal that can alter the chemical reaction of the irrigant solutions, the pressure inside the root canal system cannot be increased. However, it is feasible to accelerate cleaning by boosting the concentration and the temperature of the irrigant in the root canal area.

In the development of recent irrigant techniques that use chemical agents that can reach deeper into root canal spaces, to optimise the apical flow or lower, the surface tension was crucial to decreasing the bacterial load in the complicated endodontic space.

Endodontic treatment is completed with the obturation phase, and there are various procedures that can be used to accomplish it, particularly warm and cold techniques. However, reviewing the literature, no significant differences between warm and cold obturation techniques were present. Nevertheless, it is rational and well established that the warm filling procedures can fill the root canal space in a 3D pattern.

For example, the endodontic anatomy does not consist only of the major canal; instead, there are different anatomical configurations. Hence, most obturation materials will fail to fill these areas using cold filling techniques.

To summarise, for optimal outcomes, we must try to clean almost all of the endodontic space and then fill it almost entirely. Concurrently with guttapercha, the most typically used sealers are those based on zinc oxide, eugenol, and resin. Presently, a new generation of sealers has been released on the market, which are bioactive sealers; the basic characteristics of new sealers are reduced toxicity and the promotion of a more prominent healing ability. Regarding the modality of use, biosealers can be utilised with a single cone technique or the hot modified technique. With this modified technique, there is no need for the guttapercha to penetrate the accessory canals, as the bioceramic sealer will flow into any concealed zones. Moreover, the in vitro tests showed that the modified obturation approach allowed the sealer to extend more thoroughly inside lateral canals compared with the conventional single-cone method [13,14].

In conclusion, understanding and applying contemporary technologies and the constant development of different strategies and endodontic materials are fundamental to rendering root canal treatment safer and more efficient.

As the Guest Editors, we sincerely value and thank the reviewers for their insightful comments and the JCM team’s support. Finally, we show our gratitude to all contributing authors for their valuable input. 

## Figures and Tables

**Figure 1:**
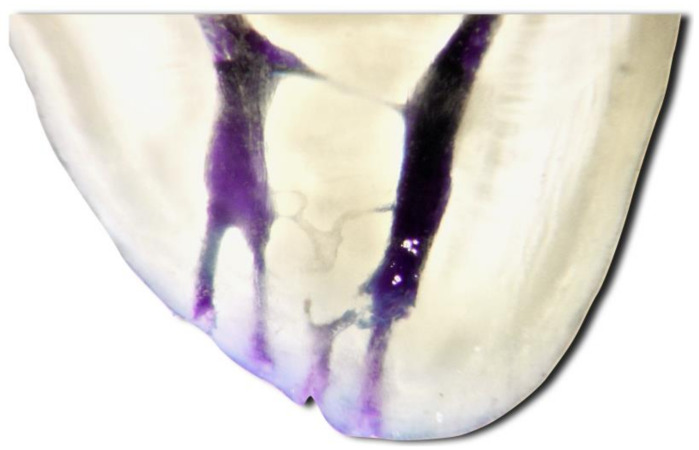
Complex apical third of the mesial root of first mandibular molar. Two deltas and multiple interconnections can be seen.

## Data Availability

Not applicable.
